# Genetic polymorphism of the N-acetyltransferase 2 gene, and susceptibility to prostate cancer: a pilot study in north Indian population

**DOI:** 10.1186/1471-2490-5-12

**Published:** 2005-08-06

**Authors:** Daya SL Srivastava, Rama D Mittal

**Affiliations:** 1 Department of Urology Sanjay Gandhi post Graduate Institute of Medical Sciences, Lucknow-226014, Uttar Pradesh, India

## Abstract

**Background:**

N-acetyltransferase 2 is phase II metabolizing enzyme that participates in the bioconversion of heterocyclic arylamines into electrophilic nitrenium ions, which are important ultimate carcinogens that are directly implicated in tumor initiation process. This study was conducted to examine; (1) whether the N-acetyltransferase 2 (NAT2) genotype is a risk factor for prostate cancer, (2) to study effect of NAT2 genotype on modifying prostate cancer risk from tobacco use.

**Methods:**

The case control study was undertaken over a period of 28 months and included 130 prostate cancer patients (CaP) and 140 controls. The NAT2 genotypes were identified by PCR-RFLP method in DNA extracted from peripheral blood. Genotype frequencies and the association of genotypes with patients and control groups were assessed by logistic regression model.

**Results:**

We observed non-significant association of rapid acetylator genotype NAT2 (OR = 1.452, 95% CI: 0.54–1.87, *P *= 0.136) in prostate cancer patients. However significant association was observed between rapid acetylator genotype NAT2 and CaP tobacco users (OR = 3.43, 95% CI: 1.68–7.02, *P*-value < 0.001) when compared with controls.

**Conclusion:**

The data suggests that the NAT2 rapid acetylator genotypes may play an important role in determining the risk of developing prostate cancer particularly in the tobacco users of north Indian population.

## Background

The human N-acetylation polymorphism is a genetic trait phenotypically allied by variation in N-acetyltransferases 2 (NAT2) activity with therapeutic agents. Acetylation polymorphism arises from the allelic variations in human arylamine N-acetyltransferases 2 resulting in the production of NAT2 proteins with variable enzyme activity or stability. Certain drugs and chemicals may contribute to the occurrence of adverse drug effects and act as susceptibility factors for certain malignancies such as prostate and bladder cancer [1,2]. NAT2 is one of the phase II enzyme that participate in the bioconversion of heterocyclic arylamines into electrophilic nitrenium ions, which are important ultimate carcinogens that are directly implicated in tumor initiation process [3]. It is expressed at high level in liver and encoded by a polymorphic gene presenting several nucleotide substitutions. Consequently the presence of the different alleles in each individual genome produce a broad range of metabolic phenotypes that vary from fully active rapid metabolizers to the less active alleles of slow metabolizers [4]. Many chemical and dietary carcinogens, such as nitrosoamines and arylamines derived from dietary fat as well as tobacco users product, acquire bioactivation and inactivation by enzymes. This suggests that polymorphism of genes encoding metabolic enzymes may represent potential risk factors [5-7].

Some studies indicated that genetically variable NATs, CYP P450 and GSTs are involved in the metabolism of drugs, carcinogens and natural products; and may be responsible for cancer susceptibility [7,8]. It has been reported that rapid acetylators genotypes of NAT2 may be at increased risk of liver and colon cancer [9], hepatocellular carcinoma [10] and colorectal cancer [11] when exposed to environmental arylamines carcinogens, due to NAT2 rapid acetylator mediated O-acetylation. Recent molecular epidemiological studies have analyzed the relationship between various metabolic enzymes, such as N-acetyltransferases (NATs) and cytochrome P450 (CYP) in etiology of prostate cancer [12,13].

It is known that human express two forms of N-acetyltransferases: NAT1 and NAT2; both genes are polymorphic. A recent review reported the nucleotide and amino-acid changes associated with various alleles and deduced phenotype from genotype. It also summarized results of molecular epidemiologic studies assessing the association of NAT1 and NAT2 genotypes with cancer risk of bladder, colon, breast, lung, head and neck and prostate [14]. A review by Chen, (2001) in prostate cancer suggests that the frequencies of some polymorphisms in certain genes differ among different racial and ethnic groups [15]. Whether these genetic variants can help explain part of the large differences in prostate cancer risk in various populations await further clarification.

The present study was undertaken to study the following objectives, i) To observe the frequencies of rapid and slow acetylators (NAT2) in CaP and control individuals ii) to study effect of NAT2 genotype on modifying prostate cancer risk from tobacco use.

## Methods

### Patient selection

The study group consisted of 130 prostate cancer patients mean age (63.3 ± 9.9) and 140 normal healthy controls mean age (56.7 ± 13.9). The criteria for the patient selection was based on clinical proforma, pathological, and histo-pathological records from the outpatient department of Sanjay Gandhi postgraduate institute of medical science, Lucknow. This study was approved by ethical committee of the Institute. Only histologically confirmed prostate cancer patients were included in the study. All cancer patients had higher Gleason scores (6–9) and was detected at advance stage due to lack of structured screening program under any health scheme in our country. Informed consent was obtained from each participant. The inclusion criteria for the controls were absence of any prior history of cancer or pre-cancerous lesions. Serological (prostate serum antigen, < 4 ng/dl), physical (digital rectal examination) and radiological examinations were performed in all control individuals in order to exclude the possibility of malignancy. The consumption of tobacco in any form (cigarette/ bidi smoking, chewing tobacco in beetle leaf, pan-masala/ gutka etc.) in both groups (cases and controls) was noted through a detailed questionnaire. The criteria of non-users are persons who never use tobacco related material like cigarette/ bidi smoking, pan-masala/ gutka, or chewing tobacco in beetle leaf whereas tobacco user were those who used all these carcinogenic material. These questionnaires were published in our other studies [24-26].

### PCR-RFLP and alleles genotyping

Genomic DNA was isolated from peripheral leucocytes by Proteinase -K digestion and phenol/chloroform method [16]. The NAT2 genotypes were determined using the PCR-RFLP as described previously [17]. PCR product of 1093 bp was generated by polymerase chain reaction using the following primer:

Forward 5'-TCTAGCATGAATCACTCTGC-3'

Reverse 5'- GGAACA AATTGG AC TTGG -3'.

Genomic DNA 200 ng was added to a PCR mixture, comprising 18.5 pmol of each primer, 200 μmol dNTP, 1.5 unit of Taq polymerase, and 5 μl, 10X PCR buffer (10 mmol/ml Tris HCl pH = 8.4, 50 mmol/ml KCl and 2.5 mol/ml MgCl_2_) in a total volume of 50 μl. PTC-100 thermocycler (MJ Research, U.S.A.) for polymerase chain reaction was employed. The reaction mixture was subjected to initial denaturation at 94°C for 5 min, followed by 35 cycles (94°C, 1 min), annealing (58.5°C, 1 min) and extension (72°C, 1 min). The final extension was done at 72°C for 10 min. Following PCR, 7 μl of PCR products were digested with four separate enzymes including Kpn1 for NAT2*5 allele, at 37°C for 2 hrs; Taq1 for NAT2*6 allele, at 56°C for 4 hrs; BamH1 for NAT2*7 allele at 37°C for 2 hrs; and Alu1 for NAT2*14 allele at 37°C for 2 hrs. Digested product was run on 2% agarose gel for NAT2*5, NAT2*7, NAT2*14 alleles and 3% agarose gels for NAT2*6 allele.

NAT2 have many alleles but more common alleles studied frequently are NAT2*5, NAT2*7, NAT2*14 and NAT2*6 allele as described in the present study also. However, we could not genotypes many other additional alleles of NAT2, which could be the limitation in our study.

### Estimating the frequency of rapid and slow acetylator

The variant and non-variant NAT2 alleles were recorded and rapid or slow acetylator phenotype assignments were deduced on the basis of NAT2 genotype [4]. Genotypes possessing two variant alleles (NAT2*5, NAT2*6, NAT2*7, or NAT2*14) were assigned as slow acetylator phenotype whereas others were assigned as rapid acetylator phenotype.

### Statistical analysis

Statistical analysis was done with SPSS 11.5 software program. Differences in genotype prevalence and association between case and control groups were assessed by binary logistic regression model. Odds ratios (OR) and its 95% confidence interval (CI) were obtained by summarizing data over two habit strata (tobacco users/ non-users). We evaluated age adjusted (confounder OR) and age unadjusted odds ratios, and 95% CI using logistic regression models. Univariant analysis, odds ratios, and 95% CI were used to describe the strength of association.

## Results

Comparative details of the observed frequency of different alleles are presented in (Table 1); which indicates significant interethnic variation in NAT2 genotypes in different populations. The NAT2*5 and NAT2*6 allele is most commonly present in our population and also in South Indian and Caucasian- American population but is rare in Japanese population, whereas the NAT2*14 allele is only found in African – American population.

**Table 1:** Frequency of NAT2 alleles in of north Indian control and other ethnic population.

Population	Allelic frequency of NAT2				Reference
		
	NAT2*5	NAT2*6	NAT2*7	NAT2*14	
North Indians (n = 140)	0.50	0.30	0. 25	0.0	Present study
South Indians (n = 166)	0. 22	0.37	0. 25	0.0	[20]
Caucasian-American (n = 372)	0. 45	0.28	0.02	0.0	[22]
African – American (n = 128)	0.30	0. 22	0.02	0.09	[22]
Japanese (n = 173)	0.01	0.20	0.13	–	[1]

The distribution of genotypes of NAT2 in control and cancer patients is shown in (Table 2). Higher frequency of NAT2 rapid acetylator was observed (64.6%) among the patient groups as compared to the controls (55.7%). However, this was statistically non significant (OR = 1.452, 95% CI: 0.54–1.87, *P *= 0.136).

**Table 2:** Frequency distribution of NAT2 genotypes in prostate cancer patients and controls.

Patients	NAT2 genotype		*P *– value	Unadjusted OR (95% CI)	Adjusted OR
	Slow – acetylators	Rapid-acetylators			
Controls (n = 140)	62 (44.29%)	78 (55.71%)		1.0 (Reference)	1.0
Prostate cancer (n = 130)	46 (35.38%)	84 (64.62%)	0.136	1.452 (0.54–1.87)	1.348 (0.39–2.28)

Adjusted OR = Age adjusted odds ratio.

The association between tobacco users and NAT2 genotypes are summarized in (Table 3). The OR for the rapid acetylator genotypes verses slow acetylator genotypes was 3.43 fold higher for the susceptibility of prostate cancer as compared to the controls (OR = 3.43, 95% CI: 1.68–7.02, *P*-value = 0.001) (Table 3).

**Table 3:** Association between NAT2 acetylator genotypes with tobacco users and prostate cancer patients.

Tobacco users / non -users	Controls (n = 140)	Ca-Prostate (n = 130)	*P*-value	Unadjusted OR (95% CI)	Adjusted OR (95% CI)
Non-users					
Slow – acetylator	43(42.16)	29 (42.03)		1.0(Ref)	1.0(Ref)
Rapid-acetylator	59(57.84)	40(57.97)	0.987	1.01(0.49–2.11)	1.04(0.54–2.03)
Tobacco Users					
Slow – acetylator	19 (50%)	17(27.87)	0.492	1.33(0.49–2.47)	1.45 (0.61–3.46)
Rapid-acetylator	19 (50%)	44(72.13)	0.001	3.43(1.68–7.02)	4.37(2.02–9.45)

Adjusted OR = Age adjusted odds ratio.

**Figure 1:**
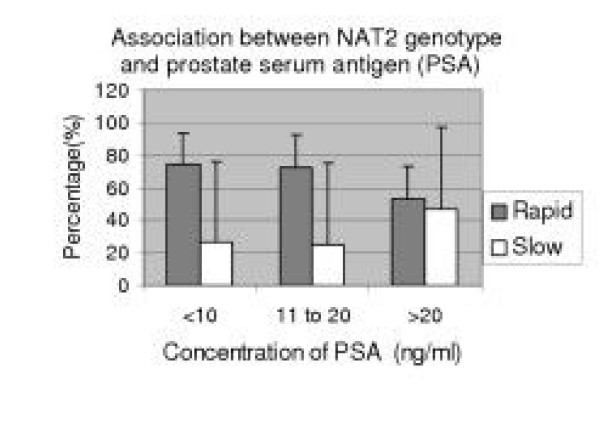
<10 = PSA (prostate serum antigen) value less than 10 ng/ml, >10 = PSA value in between 10–20 ng/ml and >20 = PSA value greater than 20 ng/ml.

**Figure 2:**
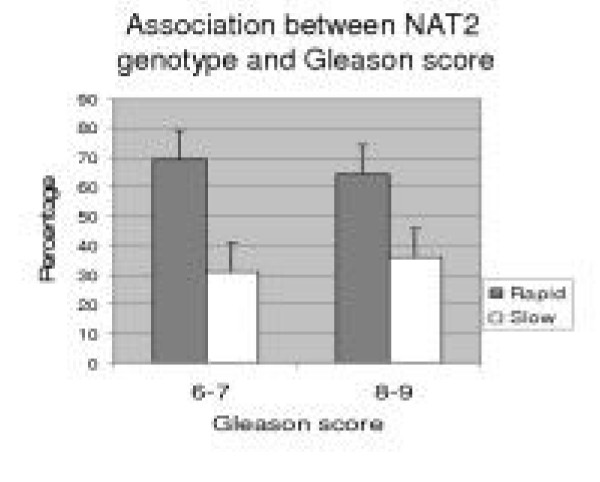
6–7 = Gleason score 6 and 7, 8–9 = Gleason score 8 and 9 of prostate tumor.

We categorized prostate serum antigen value (PSA) into three group (PSA = <10 ng/ml, >10 ng/ml,>20 ng/ml) and Gleason score into two group (GS = 6–7 and 8–9). And we observed that NAT2 genotypes were non-significant with PSA (*P *= 0.090) or Gleason score (= 0.678) for risk of prostate cancer in our population (Fig 1 and Fig 2).

## Discussion

The present NAT2 genotyping study based on molecular methods in discriminating the acetylator genotypes both in controls and prostate cancer patients is the first of its kind in north Indian population. Rapid acetylator genotypes were comparatively predominant (55.7% and 64.6%) as compared to the slow acetylator genotypes (44.3% and 35.4%).

The results observed in the present study suggest, that NAT2 genotype has a trend of association for prostate cancer risk when considered alone (OR = 1.452, 95% CI: 0.54–1.87, *P *= 0.136) but is statistically non-significant (Table 2). However, no association could be established between NAT2 genotype and PSA and/or Gleason score (Fig 1 and Fig 2). Our findings agree with previous studies that showed significant association of prostate cancer for NAT2 rapid acetylator genotype in American study (23) whereas non significant association was observed in Swedish, Danish [12]) and Spanish population [13]. On the contrary, there was significant association reported with slow acetylator genotypes of NAT2 in Japanese population [1]. The discrimination in association study from our observation could be related to ethnic variation.

However, in the present study we observed significant association between the NAT2 genotypes in tobacco users as compared to the controls (Table 3). The synergistic interaction between the rapid acetylators genotype with tobacco users obtained in our study implies that exposure to tobacco, activates heterocyclic amines that are substrates for NAT2 which may increase the risk for prostate cancer. These observations are in agreement with the reports published by the investigators in liver and colon. In the liver heterocyclic amines may be N-hydroxylated by the hepatic CYP1A2, and in turn O-acetylated by NAT enzymes to an active form that can develop DNA adducts [5,9]. NAT2 genotypes studied in hepatocellular carcinoma [10] and colorectal cancer [11] have indicated the prevalence of rapid acetylator among patient population. Thus, the present study suggests that rapid acetylator genotype could be associated with the susceptibility to prostate cancer especially in tobacco users. The mechanisms behind this association indicate that the slow acetylator genotypes should decrease the generation of critical intracellular concentration of such ultimate carcinogens, and thus reduce tumorogenesis upon environmental exposure (tobacco users). However, rapid acetylator genotypes should increase the generation of such ultimate carcinogens and enhance tumorogenesis by the pathway of O-acetylation. The results found in the present study reveal a markedly increased frequency of allele encoding the active genotype, among the patients cohort that entirely fit the above model, and are consistent with genotype / phenotype based studied that demonstrate an excess of rapid acetylator among the prostate cancer patients.

In the controls, slow allele of NAT2 is present up to 90% in Arab population [21], 40–60% in Caucasians including Indian [18] and 5–25% East Asian [19]. We observed 44% of slow acetylator genotypes; however, another study from South Indian population indicated 74% of slow acetylator genotype [20]. Thus it indicates that frequency of slow allele observed in our population matched with studies in Caucasians population [18]. Differences of distribution of slow allele of NAT2 between our and south Indian population is due to the different ethnic /or geographical environment.

## Conclusion

In conclusion, this study indicates that NAT2 rapid acetylator genotype exhibit a trend of association with the risk of developing prostate cancer, and more so in case of patients who are tobacco users.

## Competing interests

The author(s) declare that they have no competing interests.

## Authors' contributions

DSL Srivastava carried out the molecular genetic studies, participated in analyzing the data & drafted the manuscript. RD Mittal participated in designing of the study & manuscript. All authors read and approved the final manuscript.

## Pre-publication history

The pre-publication history for this paper can be accessed here:


